# Long-read sequencing and optical mapping generates near T2T assemblies that resolves a centromeric translocation

**DOI:** 10.1038/s41598-024-59683-3

**Published:** 2024-04-18

**Authors:** Esmee ten Berk de Boer, Adam Ameur, Ignas Bunikis, Marlene Ek, Eva-Lena Stattin, Lars Feuk, Jesper Eisfeldt, Anna Lindstrand

**Affiliations:** 1 Department of Molecular Medicine and Surgery, Karolinska Institutet, 171 76 Stockholm, Sweden; 2 Department of Clinical Genetics and Genomics, Karolinska University Hospital, 171 76 Stockholm, Sweden; 3 Science for Life Laboratory, Karolinska Institutet Science Park, 171 65 Solna, Sweden; 4 Department of Immunology, Genetics and Pathology, Uppsala University, 752 36 Uppsala, Sweden

**Keywords:** Computational biology and bioinformatics, Genetics

## Abstract

Long-read genome sequencing (lrGS) is a promising method in genetic diagnostics. Here we investigate the potential of lrGS to detect a disease-associated chromosomal translocation between 17p13 and the 19 centromere. We constructed two sets of phased and non-phased de novo assemblies; (i) based on lrGS only and (ii) hybrid assemblies combining lrGS with optical mapping using lrGS reads with a median coverage of 34X. Variant calling detected both structural variants (SVs) and small variants and the accuracy of the small variant calling was compared with those called with short-read genome sequencing (srGS). The de novo and hybrid assemblies had high quality and contiguity with N50 of 62.85 Mb, enabling a near telomere to telomere assembly with less than a 100 contigs per haplotype. Notably, we successfully identified the centromeric breakpoint of the translocation. A concordance of 92% was observed when comparing small variant calling between srGS and lrGS. In summary, our findings underscore the remarkable potential of lrGS as a comprehensive and accurate solution for the analysis of SVs and small variants. Thus, lrGS could replace a large battery of genetic tests that were used for the diagnosis of a single symptomatic translocation carrier, highlighting the potential of lrGS in the realm of digital karyotyping.

## Introduction

Structural variants (SVs) refer to genetic alterations spanning more than 50 base pairs (bp). SVs play an important role in phenotypic diversity but may also represent pathogenic disease-causing variants or strong genetic risk factors. For a comprehensive understanding of a specific event and its associated clinical consequences, it is essential to pinpoint the position of breakpoint junctions (BPJs) and resolve the structure of the derivative chromosome^1^. However, even though the current clinical testing methodologies can capture a wide range of variant types and sizes they have limitation in precision and resolution, and the detailed genomic architecture of clinically relevant SVs often remains unresolved.

There are currently many methodologies used in routine clinical diagnostics for characterization of SVs and chromosomal aberrations. Karyotyping comprehensively captures large variants sized above 5–10 mega bases (Mb)^2^, but misses smaller variants due to limitations in resolution and lacks the precision to capture BPJs at the nucleotide-level. Chromosome microarray analysis (CMA) increases the resolution for copy number variation (CNVs) to 1–100 kb but does not detect balanced variants^3^. Short-read genome sequencing (srGS) can detect both CNVs and balanced variants and characterize BPJs with nucleotide resolution^4^. However, srGS has limitations such as problems with phasing breakpoints of complex genomic rearrangements and with accurate mapping in repetitive regions^5^, problems that the longer reads provided by lrGS or optical mapping often resolve^6^. Furthermore, lrGS can be used directly for a de novo assembly of contigs. This enables large-scale detection of SVs, the discovery of novel insertions and a better resolution of repetitive regions across the genome^7^. At the same time, using de novo assembly for SV calling minimizes reference bias^8^. Additionally, the latest lrGS technologies support methylation analysis, paving the way to detect imprinting disorders and disease associated methylation signatures. In this way, lrGS has the potential to not just replace but also enhance current clinical testing strategies.

We report the findings of lrGS analysis using a single SMRT cell on the Pacific Biosciences Revio System (PacBio Revio), of a symptomatic individual with a balanced centromeric translocation, t(17;19)(p13;p11)^9^. Given the challenges of detecting balanced SVs with srGS, especially when breakpoints fall within difficult-to-map regions^9,10^, we explore the ability of lrGS to accurately characterise this chromosomal rearrangement and find the exact centromeric breakpoint. By integrating the lrGS data with optical genome mapping we achieve highly continuous assemblies that surpass hg38 and require the telomere-to-telomere CHM13 (T2T-CHM13)^11^ assembly for an accurate analysis.

## Methods

### Short-read sequencing

In brief, genomic DNA derived from whole blood was sequenced to 30X depth on the HiSeqXTen (Illumina, San Diego, CA, USA) at the National Genomics Infrastructure (NGI) (SciLifeLab, Stockholm, Sweden) using the PCR-free paired-end (PE) protocol for srGS. Data was processed as described previously^12^.

### Optical genome mapping

Optical genome mapping was performed on a genomic DNA sample from the proband using dual enzymes (BspQI, BssSI) on the Saphyr platform (BioNano Genomics, San Diego, CA, USA). Data was processed as described previously^12^.

### HiFi long-read sequencing and de novo assembly

High molecular weight DNA was extracted from cultured cells using the Nanobind CBB kit (Pacific Biosciences, Menlo Park, CA, USA) according to manufacturer’s protocol. The genomic DNA was sequenced on the Revio platform (Pacific Biosciences, Menlo Park, CA, USA) at the National Genomics Infrastructure (NGI) at SciLifeLab (Uppsala, Sweden). The sequencing was performed based on standard protocols. The depth of coverage of the sequencing run was computed using mosdepth (v.0.3.3)^13^ using default options as:

- *Mosdepth* < *prefix* >  < *input bam* > 

And then collected using MultiQC (v.1.12)^14^.

The resulting data was assembled using the Hifiasm assembler (v.0.19.5)^15^ using default options as:

- hifiasm -o < Prefix > -t 32 < compressed fq file > 

This produces three files, one primary assembly as well as two phased assemblies. The Assemblies were mapped to the reference genome using minimap2 (v.2.24)^16^. Minimap2 was run as:

- minimap2 -t 8 -ax asm5 < Reference >  < Assembly >  >  < Prefix > .sam

Where the reference is either hg38 or T2T-CHM13. Alignments were sorted and converted to bam using samtools^17^. Quality measures of various de novo and hybrid assemblies were obtained using QUAST^18^. QUAST was run as follows:

- quast.py -o < output folder > -r < GRCh38 reference > –threads 16 –large < assembly > 

The assembly coverage of reference genomes was computed using TIDDIT (v.3.6.0)^19^. TIDDIT was run as:

- tiddit –cov -o < prefix > -z 1000 –bam < prefix > .sort.bam

This produces a bed file with coordinates and average coverage for each region of 1000 bp.

Genes that are not fully covered by the assembly were then counted. Reference annotations were obtained for hg38 via Gencode (v.43) and T2T-CHM13 via the human pangenome consortium (accession code GCA_009914755.4). Coordinates of protein-coding genes were extracted and then intersected with regions with coverage < 1.0 using bedtools intersect^20^. The total number of intersecting features was then counted.

### Hybrid assembly

Hybrid assemblies were created by scaffolding the Hifiasm assemblies using Bionano CMAPs, using the hybridScaffold pipeline provided by Bionano solve. The command was run as follows:

- perl hybridScaffold.pl -f -n < de novo assembly fasta > -b < BioNano cmap > -c < config > -o < output folder > -r < path to aligner > -N 1 -B 1

### Variant calling

Variant calling for the PacBio Revio contigs was performed using htsbox (htslib lite-r346, htsbox r345)^17^. HTSBox was run as follows for both hg38 and T2T-CHM13 as a reference:

- htsbox pileup -f < Reference > -c < prefix > .sort.bam >  < prefix > .sort.vcf

For both the short-reads and the HiFi long-reads, variant calling was performed with DeepVariant (v. 1.4)^21^ using standard parameters.

The RTG Tool vcfeval (v.3.12.1) was used to compare SNVs between the generated srGS and lrGs data^22^. The parameters–squash-ploidy and –bed-regions were used. The bed-regions file was created by extracting the coordinates of all exons of protein-coding genes as annotated in the Gencode annotation of hg38 (v. 43).

### Ethics declaration

The study was conducted in accordance with the Declaration of Helsinki and approved by the Regional Ethical Review Board in Stockholm, Sweden. Written informed consent has been obtained from the patient to publish this paper. The data presented in the paper has been deidentified.

## Results

### Long-reads from a single SMRT cell can be de novo assembled into large contigs

**Figure 1:**
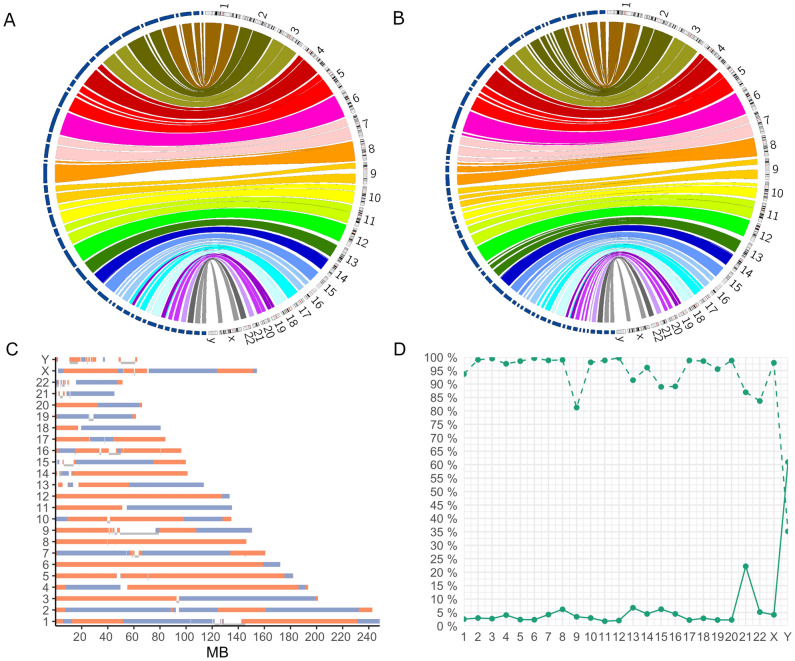
De novo assembly of HiFi long-reads yields highly continuous assemblies that nearly cover the reference genome. (**A**, **B**) Circos plot showing the alignment of de novo, hybrid assembled (**A**) and phased, de novo assembled (**B**) contigs of minimum size 5 Mb to the T2T-CHM13 reference genome. Contigs are shown in blue, the link colour is chromosome dependent. (**C**) Alignment between de novo assembly and T2T-CHM13: contigs are coloured in either blue or orange, and a gap between contigs indicates no alignment between the de novo assembly and the T2T-CHM13reference. A grey bar on the side of the gap indicates a contig spans over the alignment gap. X-axis in mega base pairs (MB) (**D**) Line chart with coverage information of the T2T-CHM13 reference genome. The dashed line indicates the percentage (%) of the reference chromosomes is covered by a contig in the de novo assembly. The continuous line indicates the percentage (%) of genes not fully covered by the de novo assembly per chromosome. Of note, the number of protein-coding genes on chromosome 21increase with 71 in T2T-CHM13, showing the clinical value of improving the assembly of acrocentric chromosome with a large fraction of hard-to-assembly regions.

The long-read genome sequencing run generated a total of 106 gigabases of data resulting in a median coverage of 34X (Supplementary Fig. 1) First, we assessed the quality of the de novo assembled HiFi long-reads using QUAST. We note that de novo assembly results in assemblies with high continuity, as indicated by high N50s and NG50s in combination with a low number of contigs (Table 1, Fig. 1A, B). If reads are phased prior to de novo assembly, the assembly length is slightly reduced but the base quality remains the same (Table 1).

**Table 1:** Assembly statistics of phased and non-phased de novo and hybrid assemblies using the PacBio Revio long-read sequences.

	Assembly size	N50	NG50	Genome fraction (%)	INDELS per 100 kbp	n contigs
de novo assembly hap 1	3,006,045,134	57,208,915	55,503,905	95.735	30.08	389
de novo assembly hap 2	2,991,479,136	42,895,890	42,895,816	95.746	29.79	386
de novo assembly no hap	3,096,550,045	62,387,384	62,387,384	97.724	29.99	270
Bionano HA hap 1	2,943,340,118	91,976,057	86,288,649	95.539	26.95	89
Bionano HA hap 2	2,937,271,221	81,016,669	78,379,872	95.613	26.54	97
Bionano HA no hap	3,024,216,483	92,473,105	92,473,105	97.647	26.76	70

We then assessed the alignment between the de novo assembly and the T2T-CHM13 reference genome. The alignment shows 50 gaps; however, the majority of these gaps are spanned by a contig that aligns on both sides of the gap (Fig. 1C). This suggests that many gaps are caused by a lack of alignment rather than a lack of sequence. In hg38 we find 240% more alignment gaps (120 gaps), especially around centromeres and on the p-arms of acrocentric chromosomes (Supplementary Fig. 2A).

The fraction of autosomes covered by the de novo assembly ranges between 81% and 100% when aligning the assembly to T2T-CHM13. Furthermore, the majority of the 21,210 protein coding genes are fully covered by the de novo assembly. Only 3.8% (800 genes) displayed less than 100% coverage (Fig. 1D). These numbers are similar in hg38 (Supplementary Fig. 2B) Notably, the count of genes that are not fully covered per chromosome appears to correlate with the chromosome’s gene density (Fig. 1D), with the exceptions of chr21 and chrY.

Next, to evaluate the added value of optical genome mapping, we made a hybrid assembly using the de novo assembly and optical mapping data. In this way, the N50 and NG50 increased and the number of contigs decreased from 270 to 70(Table 1, Fig. 1A). Even so, the hybrid assembly was not necessary to pinpoint the translocation.

### Clinical summary

The reciprocal translocation between chromosomes 17 and 19, 46,XY,t(17;19)(p13;p11), was detected by karyotyping. In brief, the clinical symptoms included congenital cataract of the left eye, epileptic seizures and high-functioning autism. The detailed clinical information was reported previously together with genomic investigations, including both srGS and shallow lrGS, that identified one translocation BPJ located within the *MINK1* gene and the other within the centromere of chromosome 19, however, it was not possible to identify the exact coordinates of the second BPJ^9^.

### De novo assembly of PacBio Revio data enables finding exact breakpoints of a centromeric translocation

**Figure 2:**
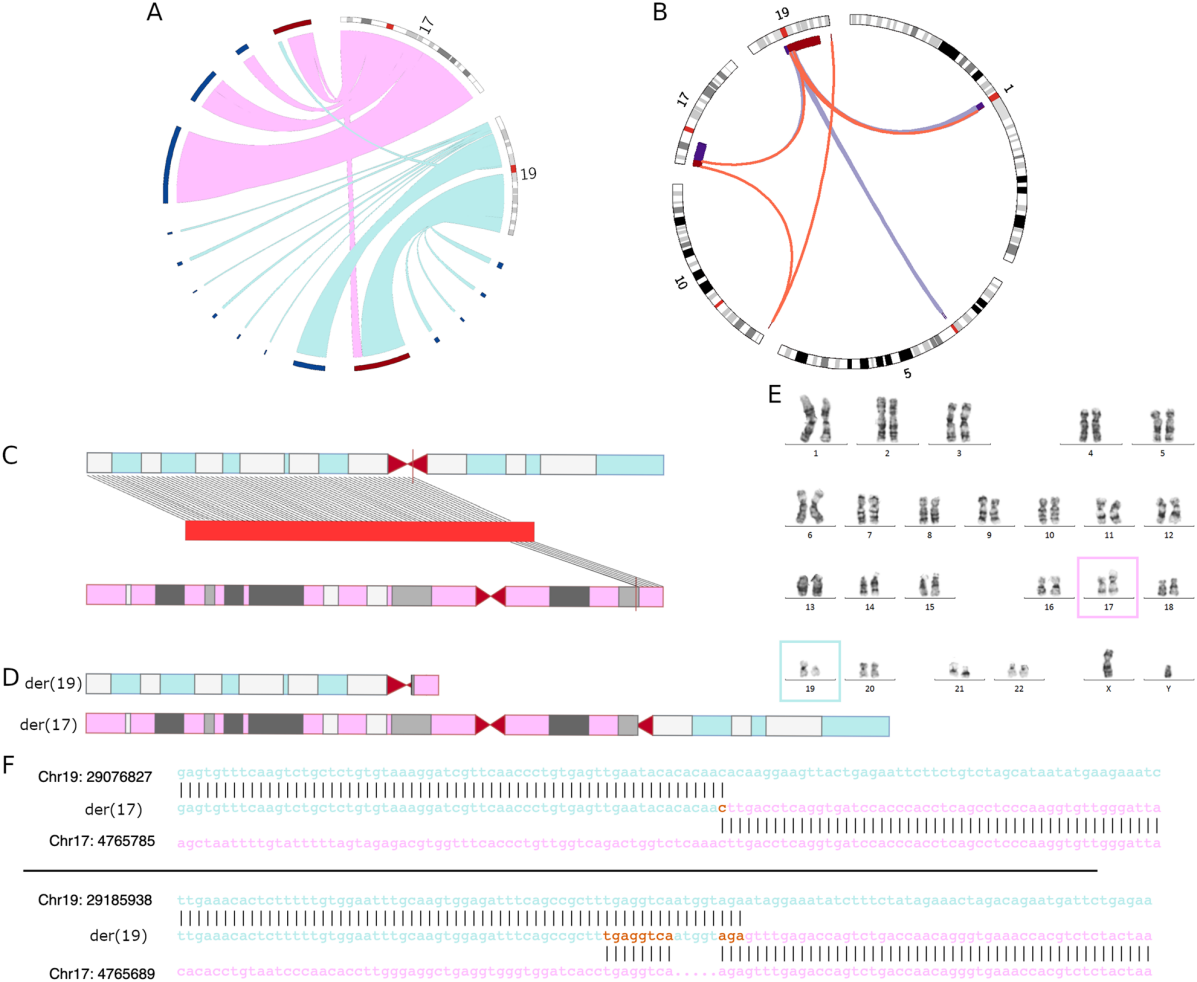
De novo assembly pinpoint the exact breakpoint within the centromere. (**A**) de novo assembled contigs aligning to chromosome 17 and chromosome 19 in hg38. Red contigs show the translocation. (**B**) Circos plot showing two contigs (From (**A**)) that show the translocation aligned to the T2T-CHM13 reference. Links indicate a translocation call, and bars indicate alignment between the chromosome and the contig. Colours represent the different contigs from which translocation calls originate. (**C**) Linear representation of one derivative (der) chromosome (red) with alignment to chromosome 19 (blue) and chromosome 17 (pink). (**D**) Derivative chromosomes coloured as in (**C**. **E**) Patient karyotype showing der(17) and der(19). (**F**) The upper panel shows the BPJ sequence of der(17), and the lower plane shows the BPJ sequence of der(19). The reference sequences are shown as each pane’s lower and upper sequences. The derivative sequence is shown in between. Horizontal lines indicate alignment. The sequence of chromosome 19 is in turquoise, and the sequence of chromosome 17 is in pink, where there is an overlap between the sequences; the sequence is shown in orange.

Using the phased de novo assembly, we identify two contigs that clearly show the translocation event (Fig. 2A). By aligning the contigs to the T2T-CHM13 reference genome, it was possible to pinpoint the exact BPJs on chromosomes 17 and 19. Both contigs show direct translocation calls between the involved chromosomes (Fig. 2B). Contrarily, using hg38 as a reference does not clearly show the correct translocation (Supplementary Fig. 3).

Subsequently, alignment to T2T-CHM13 was used to determine the exact structure and BPJ sequence of the two derivative chromosomes, der(19) and der(17) (Fig. 2C,D,E). The analysis of the translocation breakpoints identified alpha satellite repeats, SVA type B and srpRNA; however, repeats did not match between the BPJs. The BPJs contained three short stretches of microhomology of one, three and eight bp respectively (Fig. 2F). The lack of matched repeats and stretches of microhomology suggests fork-switching and template stalling (FoSTes) or microhomology-mediated break induced replication (MMBIR) as the underlying mechanism of formation^23^.

### Long-read sequencing facilitates precise detection of genetic variants

Variant calling on the primary de novo assembly with HTSBox pileup identified approximately 3.6 million SNVs (hg38) and 3.8 million SNVs (T2T-CHM13). The corresponding numbers were ~ 750.000 and ~ 720.000 for INDELS and ~ 20.000 and ~ 30.000 for SVs versus hg38 and T2T-CHM13 respectively. The large increase in SVs between hg38 and T2T-CHM13 primarily originates from repeat regions such as LINEs, satellites, LTRs and SINEs.

**Figure 3:**
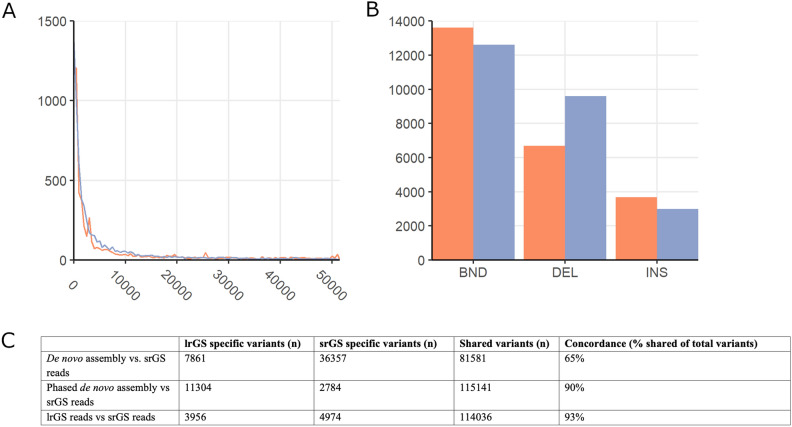
Characterisation of variants in the de novo assembly. (**A**) Size distribution of SVs called based on hg38 (Orange) and T2T-CHM13 (Blue). (**B**) Distribution of SV types based on hg38 (Blue) and T2T-CHM13 (Orange). (V) Total number of called variants when aligning the de novo assembly to hg38 and T2T-CHM13. (**C**) Comparison of the SNVs and small INDELs called between srGS and lrGS using de novo assembly, phased de novo assembly and raw reads.

Next, we compared SNV and INDEL calls in protein-coding exons between the lrGS phased assembly and the srGS reads and observe a concordance of 90% between the two methods. Both technologies find some unique variants, though most variants are shared (Fig. 3 table). Of note, when comparing the primary de novo assembly to the short read small variants the concordance becomes much lower (65%), this is due to the de novo assembly missing many variants. SNV and INDEL variants that are missed by the de novo assembly-based variant calling are usually heterozygous (Supplementary Fig. 4) and may thus be an artefact of the primary assembly lacking one of the heterozygous alleles. Furthermore, most of the variants that are missed in the non-phased de novo assembly appear within one of the haplotypes of the phased assemblies. However, some variants are missed due to a lack of alignment between the reference and the assembly (Supplementary Fig. 4D).

To assess if lrGS read-based calling performs better than de novo assembly-based variant calling we repeated the same analysis using the lrGS reads instead of the de novo assembly. Using raw reads instead of the de novo assembly leads to increased concordance (92%) (Fig. 3 table).

Finally, we detected SVs by aligning to two reference genomes, T2T-CHM13 and hg38. The called variants had a similar size distribution (Fig. 3A). However, 43.5% more deletion SVs were detected in T2T-CHM13 than in hg38 (Fig. 3B).

## Discussion

Our study demonstrates the exceptional potential of phased *de* novo assembly derived from lrGS data. We have not only been able to accurately identify and characterize a reciprocal translocation with a centromeric breakpoint but also to generate a patient de novo assembly that outperforms the standard reference genomes. Consequently, we argue that lrGS is ready to serve as a viable alternative to traditional cytogenetics.

By phased de novo assemblies we are able to directly identify and characterize a balanced translocation with one junction in centromeric sequences. Specifically, using HiFi long-reads we are able to pinpoint both breakpoints, resolve the structures of the derivative chromosomes and predict the underlying mechanism of formation (Fig. 3), without the need of any prior knowledge or other technologies. The specific translocation was previously sequenced and examined using several GS tests including short-reads, linked reads, shallow long-reads and optical genome mapping. Nonetheless, it was not possible to identify the exact centromeric breakpoint or assemble the sequences of the two derivative chromosomes^9^.

The project utilized the Revio platform that provides an economical option for long-read deep sequencing of the human genome. The decrease in cost (3 × lower than the previous Sequel II platform) will enable lrGS of many more individuals both in research and in diagnostics. Notably our de novo assembly N50 of 62.85 Mb surpass previous studies that report N50s of 15.43 to on average 35.63^24–27^, as well as higher NG50s of phased assemblies^15^. Our results are in line with a recent study which reports N50s between 20 and 100 Mb, and an average of 35.63 Mb^27^ The increase in base quality of the HiFi long-reads compared to other lrGS methods allows for more stringent alignment and overlap comparisons, which reduces the computational cost for de novo assembly compared to noisier reads^25^. Here, we show that the de novo assembly covers nearly the entire reference genome (Fig. 1C, supplementary Fig. 2), including repeat-rich regions which are often hard to assess using srGS^26^.

Given the advancement in optical mapping technology assembly contiguity could likely be further improved using newer data^28^. The hybrid assembly in this study that utilized Optical genome mapping data generated in 2016 strongly improved assembly contiguity (N50 increased by 48–89%) but newer optical genome mapping approaches are now available as well as Hi-C, which has been shown to provide high quality phased assemblies^15^. Alternatively, ONT reads can be combined with HiFi-reads to generate near telomere-to-telomere assemblies^29^. In the case presented here, hybrid assembly was not required to find the centromeric translocation but it improved quality measures. This needs to be taken into account since a hybrid approach requires multiple sequencing methods increasing both cost and laboratory complexity. Likely, in a future clinical setting, genomic tests would be conducted in a sequential manner with unsolved or partly solved cases moved to the next step.

Contrary to SV calling, the error rate for lrGS-based SNV and INDEL calling is thought to be higher than for srGS^30^. Nonetheless, in our datasets we observed a concordance of 92% when comparing small variants called by lrGS and srGS in protein coding exons, indicating that the majority of variants are called by both technologies. The differences could be explained by several factors such as misalignment of reads or incorrect base calling. The nature of these differences will need to be confirmed using standardized benchmarking methods. Nonetheless, the data in this and other studies suggests that lrGS could be used for small variant calling^31^. Simultaneously, the data also allows for accurate phasing of haplotypes and provides methylation information^32^. This makes the lrGS suitable to find many different types of potential disease-causing variants, including compound heterozygous variants, uniparental disomy, and imprinting disorders. Many of these variant types are not properly characterised by srGS, making lrGS a good first- or second-line test for individuals with genetic diseases.

Even when using lrGS, we acknowledge that variant calling will remain challenging in some regions of the genome due to a lack of alignment between the assembly and the reference genome. Some centromeres and p-arms from acrocentric chromosomes lack alignment (Fig. 1B, Supplementary Fig. 2). Consequently, variants within these regions may remain undiscovered. To address this, we propose using graph genomes for alignment to highly variable regions^33^. Both centromeres^34^ and p-arms of acrocentric chromosomes^35^ show considerable variability within and between populations. Representing dynamic regions of the genome as graph structures rather than in a linear manner increases the rate of alignment to the reference^36^. This would allow us to harness the full potential of lrGS de novo assembly and enable variant calling in all areas of the genome. Such a graph genome should preferentially be composed of lrGS sequencing of various individuals in the local population to fully capture the within population diversity of centromeres and other variable regions.

In conclusion, by sequencing a unique individual with an elusive chromosomal rearrangement, we show that with the most recent HiFi lrGS enables a de novo assembly-based approach that can resolve difficult to characterise SVs including a translocation with a centromeric breakpoint. This ability to detect SVs^26^ in combination with seemingly accurate SNV calling clearly highlights that lrGS has the potential to replace many of the current clinical genetic tests in the near future, simplifying the diagnostic workflow^37^. As such, we suggest that lrGS is near ready to be used as a clinical test combining digital karyotyping, small variant detection and phasing as well as an epigenetic analysis. Nonetheless, to fully integrate lrGS in primary genetic diagnostics it is essential to further develop analytical tools and bioinformatic pipelines.

### Supplementary Information

Supplementary Figures.

## Data Availability

The long read genome sequencing data of the individual reported in this article will be available through the European ‘1 + Million Genomes’ Initiative that is being set up. As a clinical case data will be stored at the National Genomics Platform managed by Genomic Medicine Sweden, accession number GMS-RD_00001.
