# Supplementary material for: Long-read sequencing and optical mapping generates near T2T assemblies that resolves a centromeric translocation
Source: Sci Rep. 2024 Apr 18;14:9000. doi: 10.1038/s41598-024-59683-3 (PMC11026446; doi:10.1038/s41598-024-59683-3)
Supplement: Supplementary file 1 — Supplementary Figures. [file 41598_2024_59683_MOESM1_ESM.docx]

Long-read sequencing and optical mapping generates near T2T assemblies that resolves a centromeric translocation

Esmee ten Berk de Boer^1,2,3^, Adam Ameur^4^, Ignas Bunikis^4^, Marlene Ek^1,2^, Eva-Lena Stattin^4^, Lars Feuk^4^, Jesper Eisfeldt^1,2,3 *^, Anna Lindstrand^1,2^

**Supplementary results**

**Supplementary Figure 1**

**
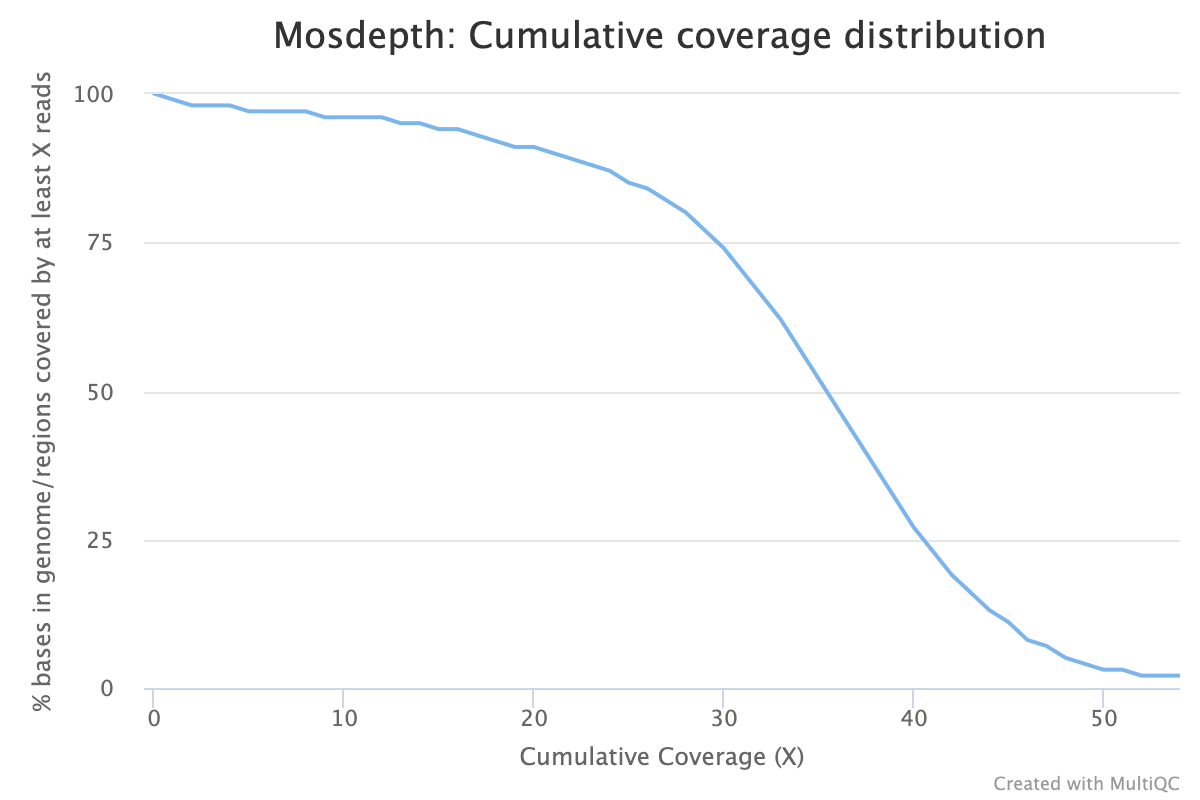
**

**Cumulative Coverage as computed by Mosdepth**.

**Supplementary Figure 2**


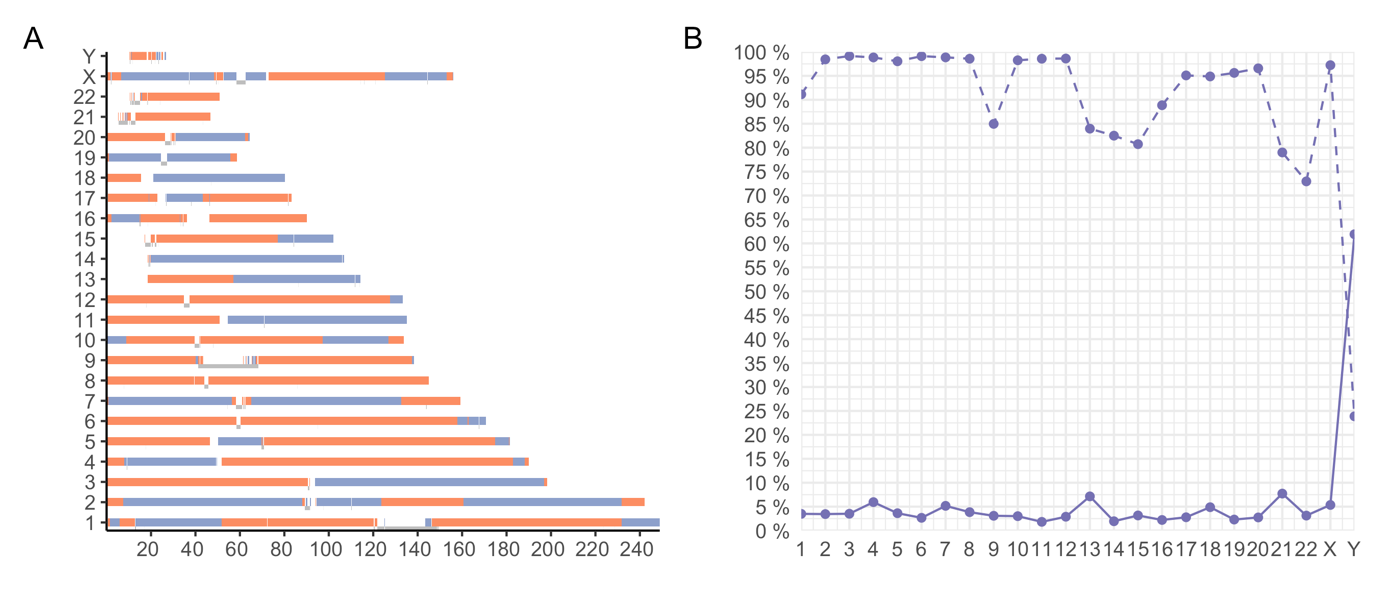


***De novo* assembly of HiFi long-reads covers the hg38 reference genome.** A) Alignment between *de novo* assembly and hg38, contigs are coloured in either blue or orange, a gap between contigs indicates there is no alignment between the *de novo* assembly and the hg38 reference. A grey bar on the side of the gap indicates a contig spans over the alignment gap. X-axis in MB. B) Line chart with coverage information of the hg38 reference genome. The dashed line indicates the percentage (%) of the reference chromosomes is covered by a contig in the *de novo* assembly. The continuous line indicates the percentage (%) of genes not fully covered by the *de novo* assembly per chromosome.

**Supplementary figure 3**


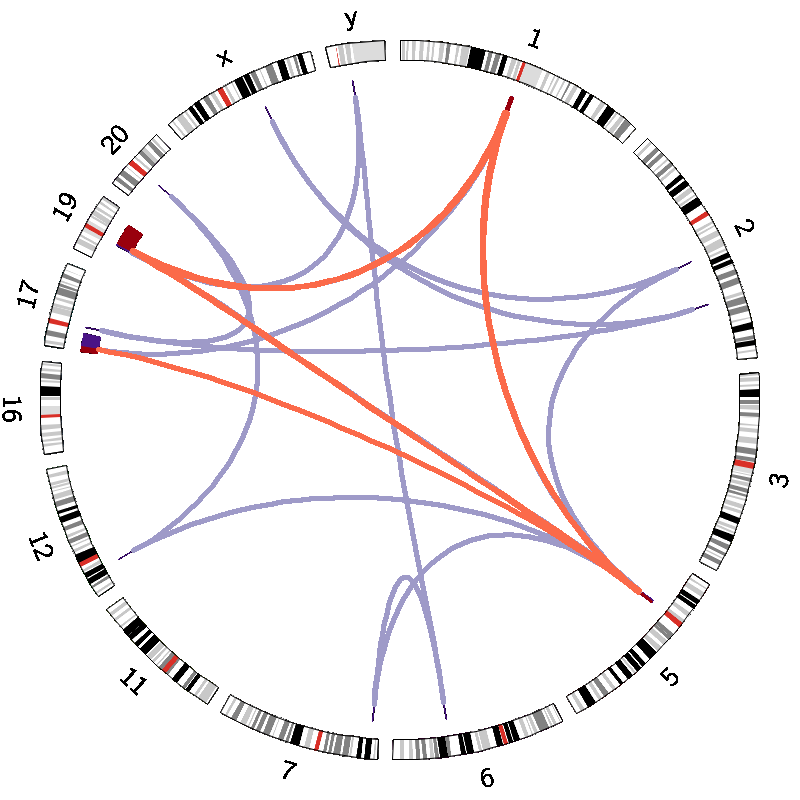


Circos plot showing two contigs (From Fig. 2A) that show the translocation aligned to the hg38 reference. Links indicate a translocation call, and bars indicate alignment between the chromosome and the contig. Colours represent the different contigs from which translocation calls originate.

**Supplementary Figure 4**


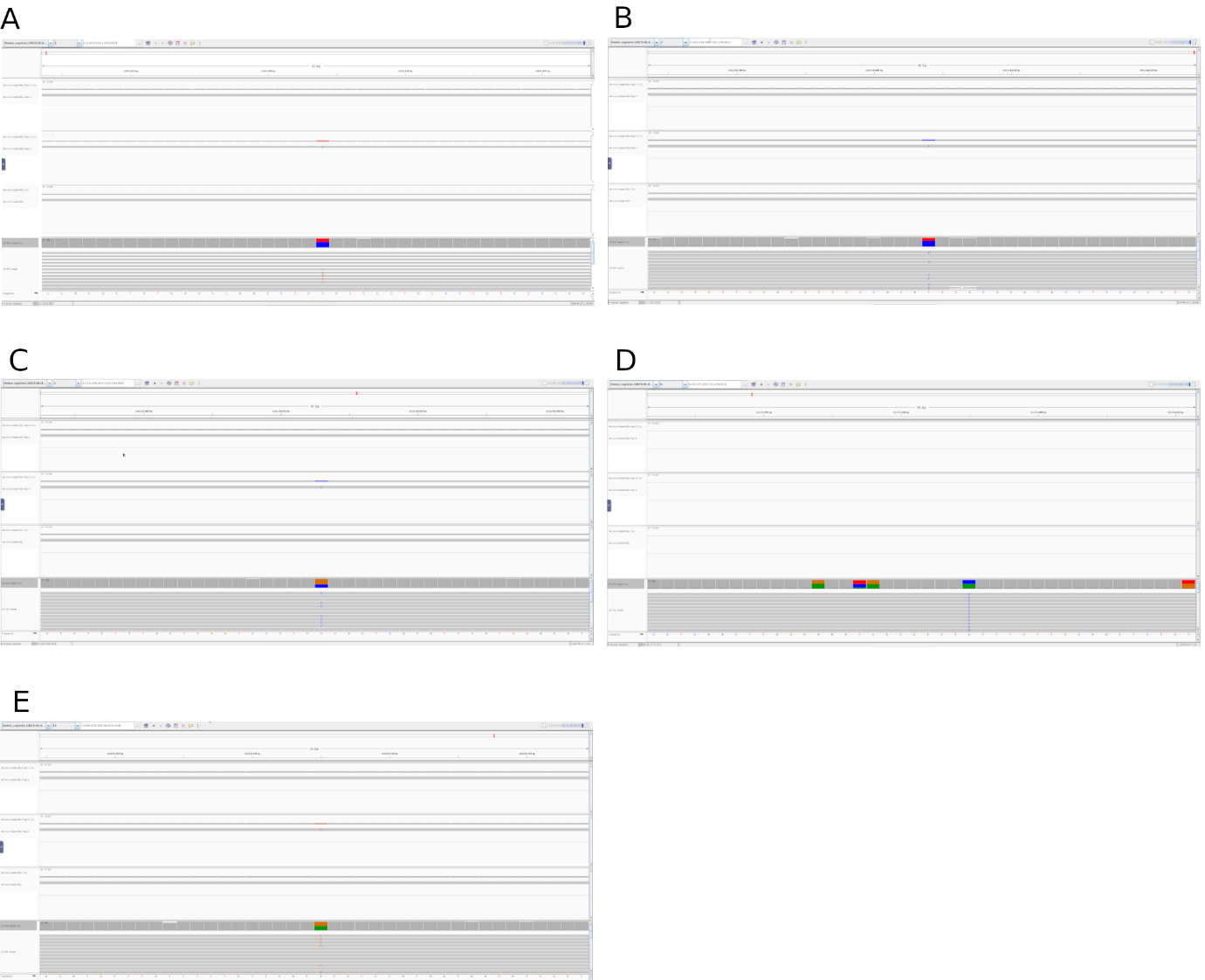


5 SNVs found using variant calling on the PacBio raw reads but not in the *de novo* assembly. For each IGV screenshot the first track is Haplotype 1 of the phased *de novo* assembly. Track two is Haplotype 2. Track 3 is the non-phased *de novo* assembly. Track four is the raw lrGS reads. Discrepancy seems to appear only in heterozygous variants. The called variant is always present within one of the phased haplotypes. A) 1:2023934. B) 2:241236604. C) 3:114236973. D) 6:32577992. E) 13:94629415.
